# Multiple-beamline operation of SACLA^1^

**DOI:** 10.1107/S1600577519001607

**Published:** 2019-02-22

**Authors:** Kensuke Tono, Toru Hara, Makina Yabashi, Hitoshi Tanaka

**Affiliations:** a XFEL Utilization Division, Japan Synchrotron Radiation Research Institute (JASRI), 1-1-1 Kouto, Sayo-cho, Sayo-gun, Hyogo 679-5198, Japan; b RIKEN SPring-8 Center, 1-1-1 Kouto, Sayo, Hyogo 679-5148, Japan

**Keywords:** X-ray free-electron laser, SACLA, multiple-beamline operation, SCSS+, photon beamline

## Abstract

The SPring-8 Ångstrom Compact free-electron LAser (SACLA) began parallel operation of three beamlines (BL1–3) in autumn 2017 to increase the user beam time of the X-ray free-electron laser. The success of the multiple-beamline operation is based on two technological achievements: (i) the fast switching operation of the SACLA main linear accelerator, which provides BL2 and BL3 with pulse-by-pulse electron beams, and (ii) the relocation and upgrade of the SPring-8 Compact SASE Source for BL1, for the generation of a soft X-ray free-electron laser. Moreover, the photon beamlines and experimental stations were upgraded to facilitate concurrent user experiments at the three beamlines and accommodate more advanced experiments.

## Abstract 2

The parallel operation of three free-electron laser (FEL) beamlines was first conducted using the SPring-8 Ångstrom Compact free-electron LAser (SACLA), to offer more opportunities for advanced studies using X-ray FELs.

## 1. Introduction   

An X-ray free-electron laser (XFEL) device uses a high-brightness electron beam to achieve a high-gain amplification of X-rays. Such an electron beam is generated solely by a linear accelerator (LINAC). Given that a LINAC can typically only drive one undulator beamline, the availability of XFELs is significantly below the satisfactory level. Beam time shortage is a common issue among XFEL facilities; therefore, the distribution of the electron beams to multiple beamlines was attempted at superconducting (Faatz *et al.*, 2016 ▸; Schloz, 2018 ▸) and normal conducting (Hara *et al.*, 2016 ▸; Reiche, 2011 ▸) LINACs. An alternative approach is the splitting of a photon beam for simultaneous experiments (Zhu *et al.*, 2014 ▸).

The SPring-8 Ångstrom Compact free-electron LAser (SACLA) began its operation in 2011 with two beamlines, namely an XFEL beamline (BL3) and a spontaneous-radiation beamline (BL1) (Ishikawa *et al.*, 2012 ▸). The two beamlines could not be operated simultaneously, given that the main LINAC directed an electron beam to either of the beamlines, as is common for most LINACs. The XFEL beamline (BL3), which almost all users requested to use, was mainly operated at the initial stage. Soon after the initial user operation in 2012, beam time shortage was recognized as a significant issue. To meet the increasing demand for longer beam times, a second XFEL beamline (BL2) was built in 2014. This beamline is operational in parallel with BL3 using a fast kicker magnet, which was installed at the end of the SACLA main LINAC (Kondo *et al.*, 2018 ▸). Two years after the initial demonstration of the parallel operation in 2015 (Hara *et al.*, 2016 ▸), the simultaneous use of BL2 and BL3 was implemented in 2017 (Hara *et al.*, 2018 ▸).

The spontaneous-radiation beamline was upgraded to a soft X-ray FEL (SXFEL) beamline (Togawa *et al.*, 2017 ▸; Owada, Togawa *et al.*, 2018 ▸). Hence, the SPring-8 Compact SASE Source (SCSS) (Shintake *et al.*, 2008 ▸), which was a test accelerator of SACLA, was relocated to the undulator hall in 2014 as a dedicated BL1 LINAC. This LINAC, which is now referred to as SCSS+, allows for the independent operation of BL1 at a frequency of 60 Hz. In 2016, the user service of BL1 was restarted to provide SXFEL.

The multiple-beamline operation of BL1, BL2 and BL3 resulted in a significant increase in the user beam time from ∼3500 h per year (before 2016) to ∼5100 h per year (2017). To facilitate the simultaneous use of the beamlines, reorganized or newly built experimental stations were used. Moreover, SACLA facilitated new capabilities to enable advanced experiments through extensive upgrades of photon beamlines and experimental instruments.

This paper presents a report on the multiple-beamline operation of SACLA. In Section 2, a discussion on the current status of the XFEL and SXFEL sources and their development for the realization of parallel operation is presented. In Section 3, the upgrades of the photon beamlines and experimental stations are discussed. The basic policies of the beam time allocation of the three beamlines are also presented. These policies are based on the multiple-beamline operation over a single year and will be updated in the future. The final part of this paper is devoted to a summary and perspectives.

## 2. FEL sources of SACLA   

### 2.1. Accelerators and undulators   

There are three key technologies used for the XFEL sources of SACLA: (i) a thermionic low-emittance electron gun, (ii) C-band accelerators and (iii) in-vacuum short-period undulators. The SACLA facility size was considerably reduced using these technologies. The periodic length of the in-vacuum undulator is 18 mm, which is approximately half the length of conventional out-of-vacuum undulators (Tanaka *et al.*, 2012 ▸). The short-period in-vacuum undulators help reduce the required electron beam energy for the XFEL operation. In addition, the C-band accelerating structures provide a 35–40 MeV m^−1^ accelerating gradient, which is higher than that of conventional S-band systems by a factor of two, for the reduction of the accelerator size (Inagaki, Kondo *et al.*, 2014 ▸).

**Figure 1:**
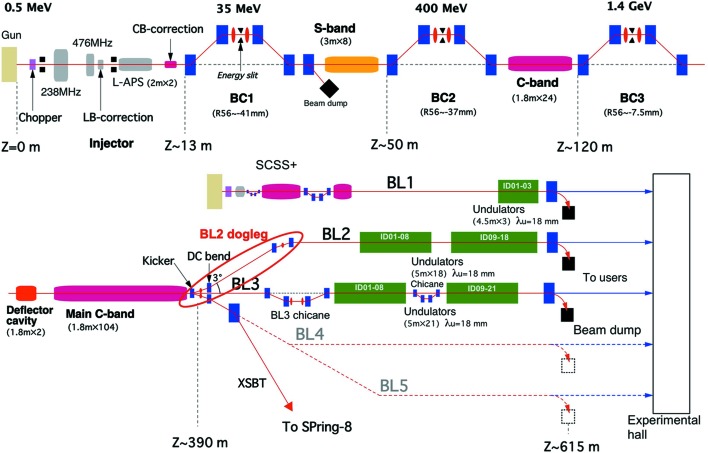
Schematic layout of the SACLA accelerator.

Fig. 1 ▸ presents a schematic view of the SACLA accelerator. The electron gun uses a thermionic cathode made from a CeB_6_ single crystal. The cathode is heated up to approximately 1500°C, and high-voltage pulses of 500 kV are applied for electron emission (Togawa *et al.*, 2007 ▸). A 1 ns electron bunch is then sliced out from the gun emission using a fast chopper, and compressed using velocity bunching in the injector (Asaka *et al.*, 2017 ▸). The electron bunch is further compressed using a three-stage bunch compressor and then accelerated to a nominal energy of 8 GeV. The nonlinearity of the bunch compression process is compensated for by two correction radiofrequency (RF) cavities in the injector, and a final peak current greater than 10 kA is achieved (Togawa *et al.*, 2009 ▸).

The undulator hall of SACLA can accommodate up to five beamlines. Three undulator lines have been installed so far for two XFEL beamlines (BL2 and BL3) and one SXFEL beamline (BL1). The magnetic gap of the in-vacuum undulators is variable; thus, the laser wavelength can be finely tuned by varying the undulator *K* value. The length of one undulator unit is 5 m, and its magnetic gap is closed to around 3 mm in the daily operation, which corresponds to a *K* value of 2.7. The number of undulator units is 3 for BL1, 18 for BL2 and 21 for BL3. Table 1 ▸ presents a summary of the major operational parameters of the three beamlines.

The duration and energy of the XFEL pulses are typically 5–10 fs (full width at half-maximum; FWHM) and 500–600 µJ at 10 keV with a pulse frequency of 60 Hz (Inubushi *et al.*, 2012 ▸; Tamasaku *et al.*, 2013 ▸), respectively. In addition to normal single-color self-amplified spontaneous emission (SASE), SACLA can also generate two-color double-laser pulses by setting the undulator gaps to two different values at BL3 (Hara, Inubushi *et al.*, 2013 ▸). The wavelengths of the two colors are independently tunable over a wide spectral range greater than 30%. For example, photon energies of 7–15 keV can be achieved within the current tunable range of the undulator (*K* = 1.5–2.7) at a standard electron-beam energy of 8 GeV. By retarding the electron bunch using a chicane installed at the middle of the BL3 undulators, the time separation between the two pulses can be adjusted with a sub-femtosecond resolution. The maximum delay between the two pulses is ∼280 fs at 8 GeV. In principle, given that the two laser pulses are generated from the same electron bunch, no time jitter occurs. Thus, the two-color operation of the SACLA offers an ideal and unique tool for the evaluation of dynamics in broad scientific fields.

The SXFEL beamline BL1 is driven by a dedicated LINAC, which runs independently from the SACLA main LINAC. The accelerator of BL1 (SCSS+) is the former SCSS test accelerator, to which six C-band accelerating structures and a second bunch compressor were added. Compared with the former SCSS test accelerator, the maximum beam energy is increased from 250 MeV to 800 MeV (Togawa *et al.*, 2017 ▸; Owada, Togawa *et al.*, 2018 ▸). The spectral range of BL1 is currently 40–150 eV, and the typical pulse energy obtained at the undulator output is 100 µJ at 100 eV.

### 2.2. Multiple-beamline operation of the main accelerator   

To accommodate the increasing number of user experiments, the parallel operation of the two XFEL beamlines, namely BL2 and BL3, has been tested since 2015 (Hara *et al.*, 2016 ▸, 2018 ▸). In the multiple-beamline operation, a switchyard installed at the end of the linear accelerator distributes the electron bunches to the two beamlines in a pulse-to-pulse manner. The switchyard is composed of a kicker magnet and two DC bending magnets (Kondo *et al.*, 2018 ▸). The kicker deflects the electron beam horizontally by either 0° or ±1.5°, and these three directions correspond to the two XFEL beamlines BL2 (+1.5°) and BL3 (0°), and an electron injection line (−1.5°) to the SPring-8 storage ring referred to as the XFEL-to-synchrotron beam transport. After the kicker magnet, the electron beam is further deflected by the DC bending magnet, as shown in Fig. 1 ▸.

For BL2, the electron beam travels through a dogleg beam transport line (BL2 dogleg in Fig. 1 ▸). For electron-beam deflection, two double-bend achromat (DBA) structures are placed at the entrance and exit of the dogleg. To suppress the coherent synchrotron radiation (CSR) effect on the electron beam, which degrades the electron beam emittance and causes beam-orbit instability, the kicker and three DC bending magnets of the BL2 dogleg are made identical with a deflection angle of 1.5°. The horizontal betatron phase advance between the two DBA structures is set as π; thus, the CSR effect is canceled at the end of the dogleg (Hara *et al.*, 2018 ▸).

**Figure 2:**
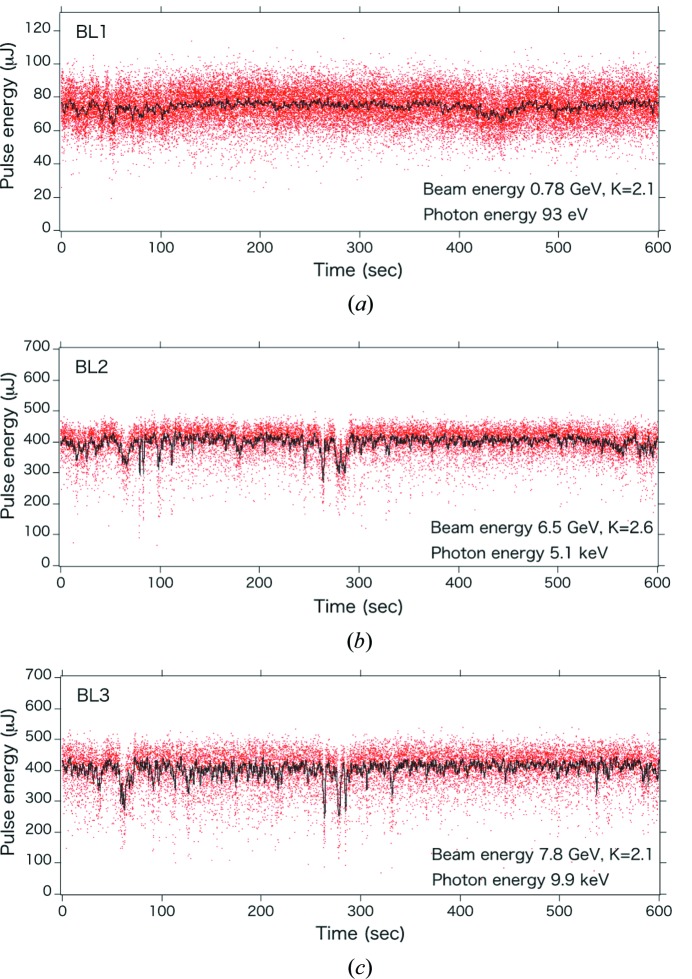
XFEL pulse energies of the three beamlines: (*a*) BL1, (*b*) BL2 and (*c*) BL3. Red dots represent single-shot results and black lines indicate averaged values over 1 s. The pulse energies of BL1 were measured after the beamline slit, and those of BL2 and BL3 were measured during the multiple-beamline operation. The electron beam energies and *K* values were 0.78 GeV and 2.1 for BL1, 6.5 GeV and 2.6 for BL2, and 7.8 GeV and 2.1 for BL3. The laser pulse frequencies were 60 Hz for BL1 and 30 Hz for BL2 and BL3.

Figs. 2 ▸(*b*) and 2(*c*) present the XFEL pulse energies obtained from the multiple-beamline operation of BL2 and BL3. The frequency of the electron beam is 60 Hz, and the electron bunches are alternately deflected to BL2 and BL3; thus, the XFEL pulse frequency is 30 Hz at each beamline.

Given that spectral tunability is one of the most important features of XFEL facilities, it should be maintained even during multiple-beamline operation. To independently tune the photon energies of the two beamlines according to user requests, the electron beam energies of the two beamlines are changed in addition to the undulator *K* values. In the case shown in Fig. 2 ▸, for example, the electron bunches are alternately accelerated to 6.5 GeV and 7.8 GeV by running several C-band accelerating structures at half the frequency of the electron beam, and then the switchyard distributes the low-energy bunches to BL2 and the high-energy bunches to BL3 (Hara, Tamasaku *et al.*, 2013 ▸). With this multi-energy operation of the LINAC, the tunable spectral range of the multiple-beamline operation is significantly enlarged (4–15 keV in a standard operation mode).

**Figure 3:**
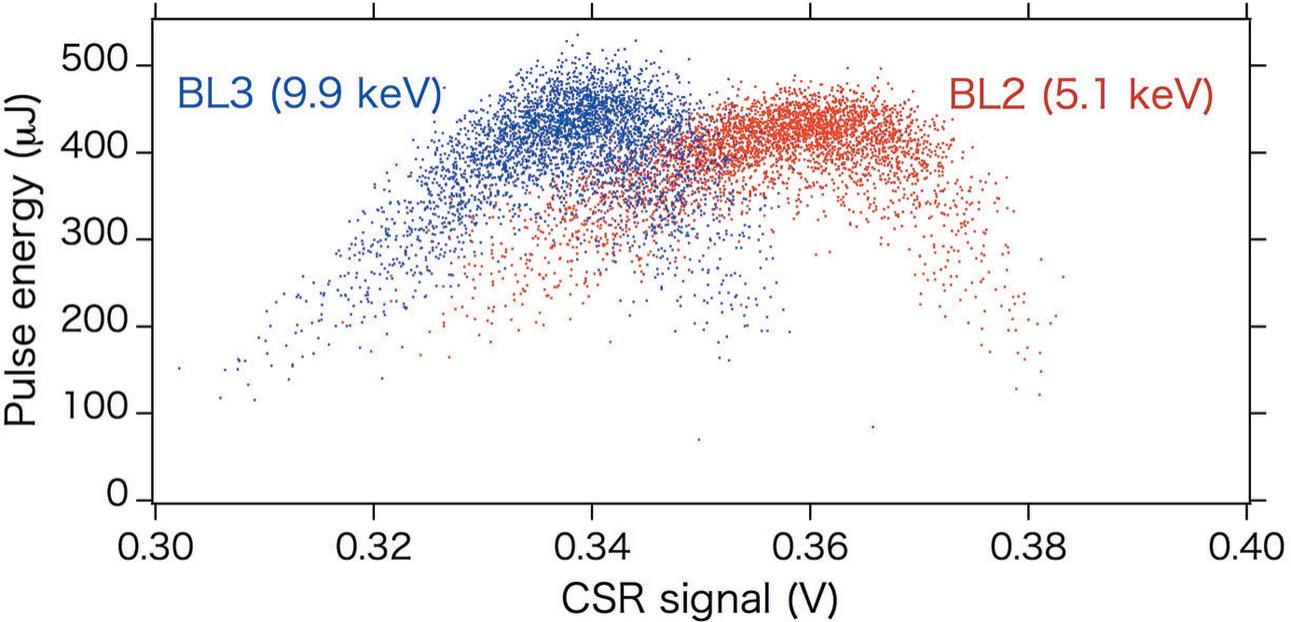
XFEL pulse energies of BL2 and BL3 plotted as a function of the CSR intensity measured at BC3. Red and blue dots represent the data of BL2 and BL3, respectively.

When operating the two beamlines in parallel, the optimum electron bunch parameters at the end of the accelerator, particularly the electron bunch length, may differ slightly for the two beamlines, to maximize the laser intensity. This is mainly due to the different momentum compaction factors (*R*_56_) of BL2 and BL3. The laser pulse energies of BL2 and BL3 were plotted with respect to the CSR intensity measured at the final compressor (BC3 in Fig. 1 ▸), which is related to the longitudinal distribution of the electron bunch, as shown in Fig. 3 ▸. It is clear that the optimum bunch lengths are different for the two beamlines. For SACLA, the electron-bunch compression parameters, namely the RF phases, are changed for the two beamlines, to simultaneously obtain the maximum laser pulse energy at both beamlines.

**Figure 4:**
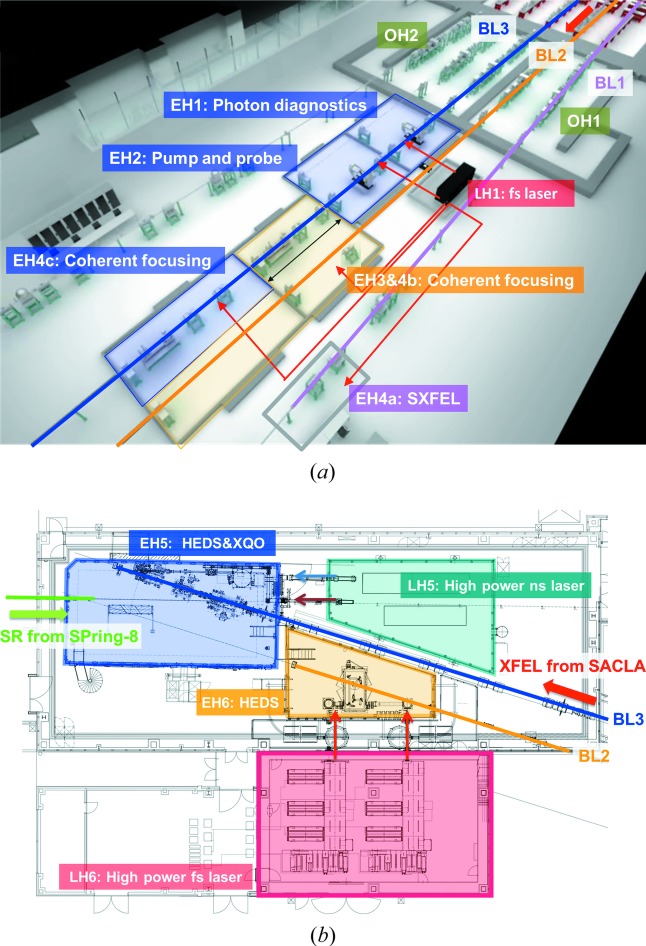
Photon beamlines and experimental hutches at SACLA: (*a*) SACLA experimental hall and (*b*) SACLA-SPring-8 Experimental Facility have six and two experimental hutches, respectively. EH: experimental hutch; OH: optics hutch; LH: laser hutch; and SR: synchrotron radiation.

In addition to the parallel operation of the two XFEL beamlines, the SXFEL beamline BL1 is independently operated using the SCSS+ accelerator [Fig. 2 ▸ (*a*)] (Owada, Togawa *et al.*, 2018 ▸). Given that the SCSS+ accelerator is triggered by the same timing system of the SACLA main LINAC, the FEL pulses of the three beamlines are synchronized. The jitter resulting from the timing system is expected to be ∼50 fs (root mean square) or smaller. Thus, the users can simultaneously use soft X-ray (SX) and hard X-ray (HX) FELs for their experiments. One possible scheme involves the use of the BL1 SX and BL2 HX beams at EH4b [Fig. 4 ▸(*a*)], for which the SX beam can be transported with total-reflection mirrors.

The three beamlines are currently in operation at SACLA, which covers a broad spectral range from extreme ultraviolet to soft and hard X-rays.

## 3. Photon beamlines and experimental stations   

This section describes the major updates from previous reports (Tono *et al.*, 2017 ▸; Tono & Hara, 2017 ▸). Fig. 4 ▸ presents the layout of the photon beamlines and experimental hutches (EHs) (Yabashi *et al.*, 2017 ▸). The major roles of the photon beamlines and EHs are summarized in Table 2 ▸.

At the SXFEL beamline (BL1), new components were installed for pulse arrival-time monitoring with a resolution of the order of 10 fs. A cross-correlation technique was employed in the arrival-time monitor, by which the SXFEL-induced change in the optical reflectivity of GaAs is probed by the femtosecond optical-laser pulses. Details of this monitoring system are described in separate articles (Owada, Nakajima *et al.*, 2018 ▸; Owada *et al.*, 2019 ▸). This beamline has one EH (EH4a), which includes focusing mirrors in the Kirkpatrick–Baez (KB) geometry and a femtosecond optical laser system. Major applications with respect to the BL1 include experiments conducted for the observation of nonlinear optical processes in gaseous atoms and molecules, and the evaluation of ultrafast phenomena in solid-state materials.

The two XFEL beamlines (BL2 and BL3) typically deliver hard X-ray pulses in the photon energy range 4–15 keV (Tono *et al.*, 2013 ▸, 2017 ▸). One of the beamlines (BL3) can extend the photon energy to ∼20 keV with a decrease in the pulse energy. Each beamline can adjust the photon energy without interfering with the experiment at the other beamline, by independently tuning the electron-beam energy in addition to the undulator *K* value. In the experimental hall, BL2 and BL3 have two and three EHs, respectively [Fig. 4 ▸(*a*)]. The two BLs were extended to the SACLA-SPring-8 Experimental Facility, where each beamline has one EH with a laser hutch, and a high-power laser system is stationed [Fig. 4 ▸(*b*)].

The first XFEL beamline (BL3) can accommodate a large variety of experiment types such as structural biology (Suga *et al.*, 2015 ▸, 2017 ▸; Nango *et al.*, 2016 ▸), ultrafast physics and chemistry (Kim *et al.*, 2015 ▸; Dean *et al.*, 2016 ▸; Albertazzi *et al.*, 2017 ▸), and X-ray quantum optics (XQO) (Tamasaku *et al.*, 2013 ▸, 2014 ▸, 2018 ▸; Yoneda *et al.*, 2015 ▸; Chumakov *et al.*, 2018 ▸). In addition to multi-purpose optics such as a double-crystal monochromator (DCM), beam-transport plane mirrors and 1 µm focusing mirrors (Ohashi *et al.*, 2013 ▸; Yumoto *et al.*, 2013 ▸; Koyama *et al.*, 2016 ▸), this beamline offers advanced optical systems such as nanometre focusing mirrors (Mimura *et al.*, 2014 ▸), a timing monitor (Katayama *et al.*, 2016 ▸), phase retarder (Suzuki *et al.*, 2014 ▸) and split-and-delay optics (Osaka *et al.*, 2017 ▸; Hirano *et al.*, 2018 ▸). The basic parameters of the multi-purpose optics are summarized in Table 3 ▸. The chicane in the undulator line allows for double-pulse operation, which produces two XFEL pulses with different wavelengths and a time separation (Hara, Inubushi *et al.*, 2013 ▸). The chicane was originally installed as a component of a self-seeding system, which is currently under development to improve its stability (Inagaki, Tanaka *et al.*, 2014 ▸; Inoue *et al.*, 2019 ▸). The advanced instruments and special operation mode are utilized, especially in experiments related to ultrafast physics and chemistry, and XQO.

The second XFEL beamline (BL2) has two experimental stations. The station with EH3&4b is mainly used for biology-oriented experiments such as protein crystallography and coherent diffraction imaging (CDI). The other station with EH6 has an experimental system for high-energy-density science (HEDS) with a high-power femtosecond laser system (Yabuuchi *et al.*, 2019 ▸). To ensure operational efficiency, the experimental apparatus is stationed at each EH of BL2 for a relatively long time period or permanently, and it is commonly used by the user groups.

A variety of experimental instruments are available at the experimental stations. Table 2 ▸ summarizes the major instruments at the optics hutches (OHs) and EHs. At each beamline, an OH contains optical and diagnostic devices that are commonly used in most experiments. Such an arrangement provides users with sufficient open space at the EH to make flexible experimental setups.

Under multiple-beamline operation, different user groups can run experiments concurrently. In such a situation, an adequate operation schedule is essential. To accommodate user requests for sufficient beam time within the capacity of SACLA, user experiments are scheduled according to the following policies.

(1) Mondays are reserved for the tuning and maintenance of the XFEL sources and photon beamlines. The other six days of the week are allotted for user experiments and instrumentation/methodology developments. In most cases, one shift (12 h) is reserved for each user beam time, for the experimental setup.

(2) At BL1, each user group requires a relatively long beam time to prepare the high-vacuum apparatus. In general, this beamline only accommodates one user group per week; thus, sufficient beam time can be provided.

(3) The coherent focusing station (EH3&4b of BL2) typically accommodates two or three user groups per week. These groups can use a standard experimental system for SFX, FPX or CDI. For users of the SFX system, a short beam time (≤6 h) can be supplied in advance for the sample screening of their main experiments. The HEDS station accommodates one group per week. The first half of the week is generally reserved for the tuning of the high-power laser system.

(4) At BL3, weekly shifts are shared by two user groups. In general, four to seven shifts are allotted to each group.

The above-mentioned policies are flexibly updated to accept new operation schemes of SACLA, which may expand the research applications, as described in the next section.

## 4. Summary and outlook   

Currently, SACLA is capable of multiple-beamline operation, which expands the research applications using XFEL. Moreover, BL1 first provided SXFEL to users in 2016. This beamline has a dedicated accelerator (SCSS+), which is operated at 60 Hz in parallel with the SACLA main LINAC. The main LINAC was successfully upgraded, and it can currently switch the electron-beam routes to BL2 and BL3 in a pulse-by-pulse manner. The first user experiments under the three-beamline operation were conducted in September 2017 without severe problems. These efforts resulted in a considerable increase in beam time. The total user beam time in 2018 reached 6000 h.

The parallel operation provides an increased beam time, and it can accommodate additional experimental schemes. For example, pump and probe measurements using SX and HX FELs are possible, given that the main LINAC and SCSS+ can be synchronized.

In the current switching operation of the main LINAC, electron bunches are transported alternately to BL2 and BL3. A new control system has been developed to allow for arbitrary pulse distribution and electron-beam injection to the storage ring of SPring-8. This is one of the most important technical requirements in the upgrade project of SPring-8 (Ishikawa *et al.*, 2014 ▸).

## Figures and Tables

**Table 1:** Major operational parameters of SACLA

	BL1 (SX)	BL2 and BL3 (HX)
Electron beam energy	800 MeV max.	8.5 GeV max.
Bunch charge	0.2–0.3 nC	0.2–0.3 nC
Peak current	0.3 kA	>10 kA
Bunch length	<1 ps (FWHM)	<20 fs (FWHM)
Repetition	60 Hz max.	60 Hz max.
Undulator period	18 mm	18 mm
Undulator *K* value	2.1 max.	2.7 max.
No. of undulator units	4.5 m × 3	5 m × 18 (BL2), 5 m × 21 (BL3)
Photon energy	40–150 eV	4–15 keV
FEL pulse energy	0.1 mJ at 100 eV	0.7 mJ at 10 keV

**Table 2:** Major instruments and applications at the beamlines (Tono *et al.*, 2013 ▸; Tono, 2017 ▸) CPA: chirped pulse amplifier; OPA: optical parametric amplifier; DAPHNIS: diverse application platform for hard X-ray diffraction in SACLA (Tono *et al.*, 2015 ▸); MAXIC: multiple application X-ray imaging chamber (Song *et al.*, 2014 ▸); OPO: optical parametric oscillator; SFX: serial femtosecond crystallography; FPX: fixed-target protein crystallography; CDI: coherent diffraction imaging; SAXS/WAXS: small-angle X-ray scattering/wide-angle X-ray scattering; HEDS: high-energy-density science; CRLs: compound refractive lenses; XRD: X-ray diffraction; fs TR-SFX: femtosecond time-resolved SFX.

Beamline	OH/EH	Major instrument	Major application
BL1 (SXFEL)	OH1	Solid attenuators, gas attenuators, intensity monitors, profile monitors, plane mirror	
	EH4a (SXFEL)	KB mirrors (<10 µm), fs laser (CPA, OPA), timing monitor	Spectroscopy, ion/electron spectroscopy, polarimetry imaging
BL2 (XFEL)	OH1	Monochromator, plane mirrors, intensity monitors, profile monitors, wavelength monitor, pulse selector†	
	EH3&4b (coherent focusing)	KB mirrors (∼1 µm), DAPHNIS, MAXIC, fs laser (CPA), ns lasers (Nd:YAG, OPO)	SFX, FPX, CDI, SAXS/WAXS
	EH6 (HEDS)	CRLs, interaction chamber, high-power fs laser	XRD, spectroscopy, SAXS/WAXS
BL3 (XFEL)	OH2	Monochromator, plane mirrors, intensity monitors, profile monitors, wavelength monitor, phase retarder, pink-beam splitter, split-and-delay optics	
	EH1 (photon diagnostics)	Pulse selector, timing monitor, monochromator	
	EH2 (pump and probe)	CRLs, fs laser (CPA, OPA)	XRD, SAXS/WAXS spectroscopy, fs TR-SFX
	EH4c (coherent focusing)	KB mirrors (1 µm), fs laser (CPA)	XRD, SAXS/WAXS spectroscopy, fs TR-SFX
	EH5 (HEDS/XQO)	KB mirrors (<0.2 µm), chamber for laser-shock experiments	XRD, spectroscopy

† Kudo *et al.* (2009 ▸).

**Table 3:** Basic parameters of the multi-purpose optics at BL2 and BL3

			Plane mirrors†				
	1 µm focusing mirrors† ‡		BL2		BL3		DCM§
Length (m)	0.6		0.6		0.5		0.09
Surface	Rh		Si		Rh/Si		Si(111)
Glancing angle (mrad)	2	3.7	2	4	2	4	<524 (<30°)
Applicable energy range (keV)	<30	<17	<15	<7.5	<33 (Rh)	<16 (Rh)	4–30
					<15 (Si)	<7.5 (Si)	
Focal length (m)	1.95 (vertical)						
	1.3 (horizontal)						

† Koyama *et al.* (2016 ▸).

‡ Yumoto *et al.* (2013 ▸).

§ Ohashi *et al.* (2013 ▸).
